# Co-occurrence of non-communicable disease risk factors among adolescents in Jos, Nigeria

**DOI:** 10.4102/phcfm.v16i1.4342

**Published:** 2024-02-15

**Authors:** Olutomi Y. Sodipo, Tolulope O. Afolaranmi, Hadiza A. Agbo, Esther A. Envuladu, Luret A. Lar, Emilia A. Udofia, Ayuba I. Zoakah

**Affiliations:** 1 Department of Community Medicine, Jos University Teaching Hospital, Jos, Nigeria; 2 Department of Community Medicine, Faculty of Medical Sciences, University of Jos, Jos, Nigeria; 3 Department of Community Health, School of Public Health, University of Ghana, Legon, Ghana

**Keywords:** non-communicable diseases, risk factors, adolescents, co-occurrence, Nigeria

## Abstract

**Background:**

The co-occurrence and clustering of risk factors for non-communicable disease (NCD) is a global public health concern.

**Aim:**

This study aimed to assess the co-occurrence and clustering of risk factors for NCDs among in-school and out-of-school adolescents in Jos North Local Government Area, Plateau State, Nigeria.

**Setting:**

Secondary schools and markets in Jos North Local Government Area.

**Methods:**

A comparative cross-sectional study was conducted among 377 in-school and 377 out-of-school adolescents, aged 10–19 years of age. An interviewer-administered questionnaire was used to collect information on behavioural and physical risk factors for NCDs. Chi-square and Mann–Whitney U tests were used for comparisons at a 5% level of significance. Statistical analysis was carried out using Statistical Product and Service Solutions (SPSS) version 23.0.

**Results:**

Of the 754 sampled adolescents, 386 (51.2%) were females and 368 (48.8%) were males. Adolescents aged 10–14 years made up 37.8% of the participants, 15 to 17 years of age accounted for 46.9% and 18–19 years 15.3%. Risk factors with the highest prevalence were a sedentary lifestyle (94.2%) and an unhealthy diet (92.4%). Majority (97.2%) had two or more risk factors while 1.9% of adolescents had no risk factor. More in-school adolescents (24.1%) had two risk factors compared to 14.1% of out-of-school adolescents (*p* < 0.001); 14.1% of out-of-school adolescents had five or more risk factors compared to 2.9% of those in school (*p* < 0.001).

**Conclusion:**

Co-occurrence and clustering of behavioural and physical risk factors was found among both in-school and out-of-school adolescents.

**Contribution:**

This study highlighted the burden of risk factors for NCDs among both in-school and out-of-school adolescents in the North-Central part of Nigeria. This is especially useful in developing targeted interventions to tackle these risk factors.

## Introduction

Non-communicable diseases (NCDs) are responsible for 74% of all deaths globally and over 75% of all NCD deaths occur in low- and middle-income countries.^1^ Majority of NCDs share several behavioural risk factors that occur together in a single individual and interact to increase the risk of NCDs and their associated complications.^2^ Tobacco use, alcohol consumption, overweight and/or obesity and mental health problems have been identified as major health risks for adolescents globally.^3^ The co-occurrence or clustering of risk factors has also been identified in adolescents across the globe.^4,5^ The World Health Organization (WHO) defines adolescents as individuals in the 10 to 19-year age group.^6^ Adolescence has been described as the period in life when an individual is no longer a child, but not yet an adult.^7^ The period of adolescence is a critical time to identify such risk factors and intervene appropriately towards improved health outcomes in adulthood.^3,8,9^ Improved health outcomes would emanate from the prevention and control of NCDs, which is critical towards the attainment of the Sustainable Development Goals (SDGs).^10^ For this to happen, there has to be concrete evidence to base interventions on. Clustering of NCD risk factors among adolescents globally as well as in some countries, including some parts of Nigeria has been documented.^4,5^ In addition, several studies have focussed on behavioural risk factors either in isolation or in clusters, leaving out physical risk factors.^11,12,13,14,15^ Hence this study aims to identify the burden of co-occurrence and clustering of behavioural and physical risk factors for NCDs among in- and out-of-school adolescents in Plateau State. Findings from this study will adequately inform public health strategies and interventions.

## Research methods and design

Research methods, design and data analysis have been previously published.^16^

### Study design

This comparative cross-sectional study was conducted between August 2020 and November 2020 among in-school and out-of-school adolescents.^16^

### Setting

Jos North Local Government Area (LGA) in Plateau State has 22 registered government schools (1 boarding school and 21 day schools) and 51 registered private secondary schools.^16^ There are eight registered markets in the LGA.^16^

### Study population and sample size

This comprised all in-school and out-of-school adolescents aged 10 to 19 years. Adolescents who attended co-educational day secondary schools were considered eligible. All adolescents who had dropped out of school without completing their senior secondary school, those who never attended school or those who participated in non-formal school programmes were considered eligible. These out-of-school adolescents had to be found in the marketplace during regular school hours.

The minimum sample size for each group was 377, calculated using the formula for a comparative study of two independent samples.

$$n=\frac{{\left(Z_{\alpha }+Z_{1-\beta }\right)}^{2}\left(p_{1}(1-p_{1})+p_{2}(1-p_{2})\right)}{{\left(p_{1}-p_{2}\right)}^{2}}$$ [Eqn 1]

A 95% confidence level was used for the study, and a *p* ≤ 0.05 was considered statistically significant. The proportion of 8.3% (*p*_1_) and 3.3% (*p*_2_) of in-school and out-of-school adolescents who smoked were obtained from previous studies.^16^

### Sampling technique

#### In-school adolescents

A two-stage sampling technique was used to select respondents from government co-educational day secondary schools. In stage one, three government co-educational day schools were selected using a simple random sampling technique by balloting from the list of the 21 registered government co-educational day secondary schools obtained from the Plateau State Ministry of Education, which served as the sampling frame. In stage two, the number of students selected from each of the three schools and the six arms in each school was done by proportionate allocation. Students were selected by simple random sampling by balloting (without replacement) using a class list containing students’ names across the various classes, which served as the sampling frame. If a sampled student did not meet the inclusion criteria, declined consent or assent, the individual personal identifier number was kept aside and another number was picked from those left by simple random sampling. This procedure was carried out in all three schools till the minimum sample size was met.^16^

#### Out-of-school adolescents

A multi-stage sampling technique was used to select respondents. In stage one, three markets were selected from the eight registered markets using a simple random sampling technique by balloting. Next, the number of registered shops to be chosen was done by proportionate allocation. Based on the assumption that at least one adolescent would be found per shop, a sampling interval for each market was calculated by dividing the total number of registered shops by the selected number of registered shops in stage three. The first shop established was obtained using simple random sampling by balloting among the shops within the sampling interval. One eligible out-of-school adolescent was selected per shop, and the questionnaire was administered. If there was more than one eligible adolescent in a shop, a simple random sampling technique by balloting was carried out to choose only one eligible adolescent. The following contiguous shop was visited if no eligible adolescent was found in a selected shop or the respondent did not consent to participate in the study. This was done until the minimum sample size was met. Research assistants were trained on obtaining informed consent or assent, good ethical conduct, content and method of questionnaire administration.^16^

### Data collection

Information on behavioural and risk factors for NCDs was collected using a pre-tested interviewer-administered questionnaire adapted from the Global School-Based Health Surveys and Global Youth Tobacco Survey questionnaires.^16^

### Measurement of variables

The independent variables were socio-demographic characteristics, while the dependent variables were the presence or absence of behavioural risk factors for NCDs computed as a composite variable and the presence or absence of physical risk factors for NCDs computed as a composite variable. A score of ‘1’ was assigned to each behavioural risk factor present, that is, current smoking, current alcohol consumption, inadequate servings of fruit and vegetables, physical inactivity and sedentary lifestyle and ‘0’ for any of the behavioural risk factors absent. Individual scores were summed up, and a total score of 1 to 5 was assigned ‘Present’ for the presence of behavioural risk factors, and a total score of 0 was assigned ‘Absent’ for the absence of behavioural risk factors.

For physical risk factors, a score of 1 or 0 each was assigned for the presence or absence of overweight or obesity and hypertension. A total score of 1 or 2 was assigned as ‘Present’ for physical risk factors, and 0 was assigned as ‘Absent’ for the absence of any physical risk factor.^16^ The blood pressure (BP) of each adolescent was measured after at least 5 min of rest in a sitting position using a digital sphygmomanometer with an appropriately sized cuff, that is, a cuff with an inflatable bladder width that was at least 40% of the arm circumference at a point midway between the olecranon and the acromion. Two readings (in mmHg) were taken 1 min–2 min apart and the average calculated.^17^ Weight was measured with a portable bathroom weighing scale (spring scale). The weighing scales were standardised by ensuring that they were calibrated to the zero mark before each use.

The weighing scale was placed on a flat surface. Adolescents were asked to remove their slippers, shoes and heavy clothing such as cardigans and belts (if any) and stand straight on the scale for measurements.^18,19^ Paired measurements were taken by two research assistants using the same weighing scale, and the average weight obtained was recorded to the nearest kilogram (kg). Height was measured with a portable and non-stretchable measuring tape. Adolescents were asked to remove their shoes and head gear, if any, and stand straight against a wall, with their heels touching the wall and face the research assistant, looking straight ahead. Paired measurements were taken by two research assistants using the same measuring tape and the average height obtained was recorded to the nearest 0.1 m.^18,19^

### Operational definitions

#### Tobacco use

Smoking any number of cigarettes in the last 30 days was considered current smoking; one who had not smoked at all was considered non-smoking.^16^

#### Alcohol consumption

Consumption of any form of alcoholic drink in the last 30 days was considered as current alcohol consumption; one who had not consumed any alcoholic drink at all was considered as non-alcohol consumption. A drink was defined as a bottle, one glass of wine or a shot of any spirits, for example, gin or red wine.^16^

#### Physical inactivity

Physical inactivity was defined as engaging less than 5 days a week with at least 60 min of moderate to vigorous physical activity daily.^16^ Examples include stretch exercises, sit ups, weight lifting, walking or riding a bicycle.^20,21^

#### Unhealthy diet

Unhealthy diet was defined as consuming less than five servings of fruit and vegetables daily. One serving of fruit was one medium-sized apple, banana or orange, and one cup of freshly squeezed fruit juice; for chopped fruit, one serving was equal to one 250 mL cup. One serving of vegetables was one cup (250 mL) of green leafy vegetables or vegetable salad.^16^

#### Sedentary behaviour

Sedentary behaviour was assessed by total screen time (sum of daily television, computer and video game time) on weekdays and the weekend. Adolescents with more than 2 h per day in front of the screen were considered to have this risky behaviour.^16^

#### Overweight

Body mass index (BMI) for age 85th to < 95th percentile (using the BMI for age percentile chart for girls and boys).^16^

#### Obesity

Body mass index for age ≥ 95th percentile (using the BMI for age percentile chart for girls and boys).^16^

#### Blood pressure

For adolescents aged < 13 years of age, BP percentiles based on gender, age and height were determined using the BP levels for age and height for boys and girls. The definitions of BP categories and stages were from the updated BP guideline by the American Academy of Paediatrics and American College of Cardiology.^22^

### Data analysis

Data analysis was done using the IBM SPSS Statistics for Windows, version 23 (IBM Corp., Armonk, N.Y., USA). A *p*-value ≤ 0.05 was considered statistically significant for all statistical tests. Adolescents were grouped based on the WHO classification: early (10–14 years), middle (15–17 years) and late (18–19 years).^16^ Frequencies and percentages were used to assess the proportion of risk factors for NCDs. Chi-square and Mann–Whitney U tests were used to compare risk factors for NCDs among in-school and out-of-school adolescents.^16^

### Ethical considerations

Ethical clearance to conduct this study was obtained from the Jos University Teaching Hospital Institutional Research Ethical Committee (No. JUTH/DCS/IREC/127/XXXI/2238). Ethical clearance and approval for the study obtained from the health research and ethics committee and State Ministry of Education have been previously published.^16^

## Results

Socio-demographics of in-school and out-of-school adolescents has been previously published.^16^

**FIGURE 1:**
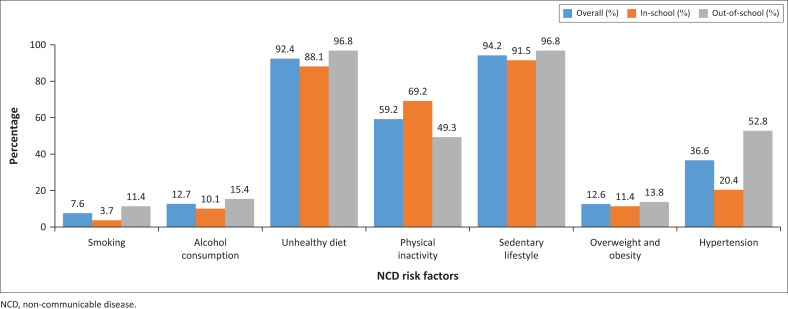
Prevalence of non-communicable disease risk factors among adolescents.

The NCD risk factors with the highest overall prevalence were a sedentary lifestyle (94.2%) and an unhealthy diet (92.4%). The lowest overall prevalence of NCD risk factors was smoking (7.6%), but out-of-school adolescents had a higher prevalence (11.4%) than in-school adolescents (3.7%). Alcohol consumption and excess weight (overweight and obesity) had a similar overall prevalence, with 12.7% and 12.6%, respectively. It was identified that 52.8% of out-of-school adolescents were hypertensive compared to 20.4% of their in-school counterparts (Figure 1).

Only 14 (1.9%) adolescents had no risk factor for NCDs while the majority (97.2%) had two or more risk factors. Over a quarter (47.1%) of all the adolescents had three risk factors. A higher proportion of in-school adolescents (91, 24.1%) had the presence of two risk factors compared to 53 (14.1%) of out-of-school adolescents, and this was statistically significant (*p* < 0.001). A higher proportion of out-of-school adolescents (53, 14.1%) had the presence of five or more risk factors compared to 11 (2.9%) of in-school adolescents, and this was statistically significant (*p* < 0.001) (Table 1).

**TABLE 1:** Co-occurrence and/or clustering of risk factors for non-communicable diseases among in-school and out-of-school adolescents.

Number of risk factors	In-school (*n* = 377)		Out-of-school (*n* = 377)		Total (*n* = 754)		*χ* ^2^	*p*
	Freq.	%	Freq.	%	Freq.	%		
No risk factor	10	2.7	4	1.1	14	1.9	2.617	0.106
One risk factor	3	0.8	4	1.1	7	0.9	0.144	0.704
Combined risk factors	364	96.6	369	97.88	733	97.2	1.223	0.269
Two risk factors	91	24.1	53	14.1	144	19.1	12.379	< 0.001*
Three risk factors	180	47.7	175	46.4	355	47.1	0.133	0.715
Four risk factors	82	21.8	88	23.2	170	22.5	0.273	0.601
Five or more risk factors	11	2.9	53	14.1	64	8.5	30.079	< 0.001*

* , statistically significant at *p* ≤ 0.05.

None of the associations between the presence or absence of risk factors for NCDs and socio-demographic variables was statistically significantly different. However, among in-school adolescents, more 18 to 19-year-olds (98.1%), more males (98.0%) and more adolescents who lived with their guardians (97.5%) had two or more behavioural risk factors for NCDs (Table 2).

**TABLE 2:** Socio-demographic factors associated with risk factors for non-communicable diseases among in-school adolescents.

Variables	No risk factor (*n* = 10)		1 risk factor (*n* = 3)		Combined risk factors (*n* = 364)		*χ* ^2^	*p*
	Freq.	%	Freq.	%	Freq.	%		
**Age group (years)**								
10–14	3	2.2	1	0.7	133	97.1	3.301†	0.464
15–17	7	3.7	1	0.5	180	95.7	-	-
18–19	0	0.0	1	1.9	51	98.1	-	-
**Sex**								
Female	3	1.5	1	0.5	166	94.9	2.834†	0.293
Male	7	4.0	2	1.1	198	98.0	-	-
**Religion**								
Christianity	10	2.7	3	0.8	353	96.4	0.869†	1.000
Islam	0	0.0	0	0.0	11	100.0	-	-
**Tribe**								
Plateau indigenous	7	2.5	2	0.7	267	96.7	0.559†	0.884
Non-plateau Indigenous	3	3.0	1	1.0	97	96.0	-	-
**Lives with**								
Parents	9	3.0	2	0.7	286	96.3	1.163†	0.504
Guardians	1	1.3	1	1.3	78	97.5	-	-
Alone	0	0.0	0	0.0	0	0.0	-	-
**Mother’s highest education**								
No formal education	3	3.4	0	0.0	85	96.6	4.726†	0.527
Primary	2	3.8	1	1.9	50	94.3	-	-
Secondary	2	1.3	2	1.3	147	97.4	-	-
Tertiary	3	3.5	0	0.0	82	96.5	-	-
**Father’s highest education**								
No formal education	2	2.4	0	0.0	81	97.6	1.971†	0.976
Primary	1	2.2	0	0.0	44	97.8	-	-
Secondary	3	2.2	2	1.5	129	96.3	-	-
Tertiary	4	3.5	1	0.9	110	95.7	-	-
**Mother’s occupation**								
Artisan	0	0.0	0	0.0	49	100.0	4.805†	0.488
Business	4	2.3	3	1.7	168	96.0	-	-
Civil servant	1	1.9	0	0.0	53	98.1	-	-
Unemployed	5	5.1	0	0.0	94	94.9	-	-
**Father’s occupation**								
Artisan	2	2.1	0	0.0	92	97.9	4.356†	0.598
Business	3	2.4	3	2.4	120	95.2	-	-
Civil servant	4	3.8	0	0.0	100	0.0	-	-
Unemployed	1	1.9	0	0.0	52	98.1	-	-

† , Fisher’s exact test.

None of the associations between the presence or absence of risk factors for NCDs and socio-demographic variables was statistically significantly different. Among out-of-school adolescents, all 18 to 19-year-olds (100%), and more adolescents who lived with their guardians (98.9%) had two or more physical risk factors for NCDs. An equal proportion of males and females had two or more physical risk factors (Table 3).

**TABLE 3:** Socio-demographic factors associated with risk factors for non-communicable diseases among out-of-school adolescents.

Variables	No risk factor (*n* = 4)		1 risk factor (*n* = 4)		Combined risk factors (*n* = 369)		*χ* ^2^	*p*
	Freq.	%	Freq.	%	Freq.	%		
**Age group (years)**								
10–14	2	1.4	1	0.7	145	98.0	1.704†	0.863
15–17	2	1.2	3	1.8	161	97.0	-	-
18–19	0	0.0	0	0.0	63	100.0	-	-
**Sex**								
Female	2	1.1	1	0.5	181	98.4	0.978†	0.858
Male	2	1.0	3	1.6	181	98.4	-	-
**Religion**								
Christianity	3	0.9	4	1.2	332	97.9	1.654†	0.576
Islam	1	2.6	0	0.0	37	97.4	-	-
**Tribe**								
Plateau indigenous	3	0.8	4	1.1	351	98.0	3.548†	0.341
Non-plateau indigenous	1	5.3	0	0.0	18	94.7	-	-
**Lives with**								
Parents	3	1.6	3	1.6	186	96.9	2.775†	0.594
Guardians	1	0.6	1	0.6	172	98.9	-	-
Alone	0	0.0	0	0.0	0	0.0	-	-
**Mother’s highest education**								
No formal education	4	1.9	2	1.0	201	97.1	4.030†	0.626
Primary	0	0.0	0	0.0	66	100.0	-	-
Secondary	0	0.0	2	2.3	85	97.7	-	-
Tertiary	0	0.0	0	0.0	17	100.0	-	-
**Father’s highest education**								
No formal education	2	1.4	2	1.4	139	97.2	2.308†	0.885
Primary	1	1.6	1	1.6	61	96.8	-	-
Secondary	1	0.7	1	0.7	144	98.6	-	-
Tertiary	0	0.0	0	0.0	25	100.0	-	-
**Mother’s occupation**								
Artisan	0	0.0	1	1.5	64	98.5	5.322†	0.350
Business	2	0.9	1	0.5	208	98.6	-	-
Civil servant	0	0.0	0	0.0	25	100.0	-	-
Unemployed	2	2.6	2	2.6	72	94.7	-	-
**Father’s occupation**								
Artisan	2	1.3	1	0.7	150	98.0	4.155†	0.645
Business	1	0.7	1	0.7	138	98.6	-	-
Civil servant	0	0.0	0	0.0	24	100.0	-	-
Unemployed	1	1.7	2	3.3	57	95.0	-	-

† , Fisher’s exact test.

## Discussion

In this study, the majority of all adolescents had at least one NCD risk factor while only 1.9% had none of the risk factors for NCDs. This was similar to findings from a study among in-school adolescents aged 15 to 18 years in Ibadan, the South Western part of Nigeria where the majority had at least one risk factor for cardiovascular disease, and only 4.6% had no risk factor.^23^ This finding was also corroborated by findings from the study among in-school adolescents aged 15 to 19 years in India where the majority had at least one risk factor and only 5.9% had no risk factor.^24^ The findings from another study among in-school adolescents aged 10 to 19 in Ibadan identified that the majority of adolescents had ever engaged in behaviours, which could predispose them to NCDs.^25^ The behaviours assessed in the study were alcohol use, tobacco use, unhealthy dietary behaviour, inadequate physical activity, but the study also assessed physical violence and sexual intercourse that are outside the scope of this current study.^25^

The findings from this current study, however, varied from those among Algerian adolescents aged 11 to 16 years of age, where 65.1% had at least one risk factor while a little over a third had no risk factor.^26^ Findings from this present study identified that the majority of all adolescents had three of more risk factors for NCDs. This was much higher than the findings from the study among in-school adolescents in Ibadan where 32.4% had three or more risk factors.^23^ Variation in findings could be attributed to the difference in the study population as well as the number and type of risk factors assessed. In the Ibadan study among adolescents aged 15 to 18 years, seven behavioural and two physical risk factors were assessed while in the Algerian study, four behavioural risk factors and one physical risk factor were assessed.^23,26^ The operational definitions for some of the risk factors assessed also varied among studies.

Majority of adolescents in this current study had at least one behavioural risk factor. This was similar to findings among university students in the South West of Nigeria, where a majority had at least one behavioural risk factor.^11^ Similarly, a study among urban slum dwellers in Kenya identified that the majority of respondents had at least one behavioural risk factor, though the proportion was lower than the findings in this present study.^27^ Clustering of behavioural risk factors was also reported in a multi-country study, that overall about a third of the 140 countries had a high burden of four or more NCD risk factors in at least 50% of adolescents aged 11 to 17 years. It was also identified in the study that the prevalence of four or more NCD risk factors increased gradually over time between 2013 and 2017.^12^ In this present study, almost a third of adolescents had three behavioural risk factors, that is, physical inactivity, unhealthy diet and sedentary lifestyle. This proportion was, however, a little lower than the findings from the study in Southern Brazil where about a fourth of the adolescents had a combination of the same risk factors.^28^ This combination of risk factors was also found to be prevalent among Vietnamese adolescents.^5^ Physical inactivity, unhealthy diet and sedentary lifestyle are three risk factors that have been found to occur together.^27,28^ Individuals who engage in sedentary behaviour such as prolonged screen time usually consume unhealthy food in the form of junk food and drinks and eat less fruit and vegetables.^27,28^ With regard to the co-occurrence of risk factors, the study conducted in Vietnam revealed that most students had at least two NCD risk factors.^5^ This was similar to a study among Brazilian adolescents where a majority had at least two behavioural NCD risk factors.^14^ The most prevalent combination of behavioural risk factors in this present study was an unhealthy diet and a sedentary lifestyle. This was similar to the findings in Tanzania among in-school adolescents where one of the prevalent co-occurrence of risk factors for NCDs was an unhealthy diet and physical inactivity.^29^ This still buttresses the interaction having a sedentary lifestyle has with the consumption of unhealthy diets.^27,28^

In this present study, a higher proportion of in-school adolescents had no risk factor for NCDs compared to out-of-school adolescents. This could be attributed to the advantage school enrolment affords those enrolled in school such as education on the dangers of engaging in health risk behaviours as well as participating in structured forms of physical activities during scheduled sports time.^30,31^ In contrast, a higher proportion of out-of-school adolescents had the presence of five or more risk factors compared to in-school adolescents, and this was statistically significant. The higher proportion of five or more risk factors among out-of-school adolescents could be attributed to stressful life events adolescents not in school go through compared to their in-school counterparts.

The state of being out-of-school and not attaining an education like one’s peers could cause such adolescents to be unhappy and depressed about their situation, hence engaging in behaviours such as smoking and alcohol consumption as an escape mechanism.^32^ It could also be that the conditions the out-of-school adolescent find his or herself are stressful, thereby leading the adolescent to smoke or drink as a means to relieve or cope with the stress. Stress and anxiety reduction have been cited as one of the main reasons for smoking.^33^ This was corroborated by findings from the study among out-of-school adolescents in a state in the South Western part of Nigeria where almost 60% of the adolescents had unstable sources of income, and this was a stressful event.^34^ Interestingly, findings from a study across seven sub-Saharan African countries did not demonstrate a statistically significant relationship with school enrolment and being overweight and consumption of unhealthy diet.^35^ A study conducted among rural adolescents in North India also did not identify a statistically significant relationship between being out of school and the presence of hypertension.^36^

In this present study, the proportion of adolescents with a combination of risk factors increased with age, though this association was not found to be statistically significant. This was similar to the findings from studies in Southern Brazil, from seven Mediterranean countries (Greece, Israel, Italy, Macedonia, Malta, Portugal, Spain) and Bangladesh, which revealed that older adolescents were significantly more likely than younger adolescents to have the co-occurrence of risk factors for NCDs.^28,37,38^ A possible explanation could be that older adolescents are usually exposed to higher stress from peer pressure and academics.^36^ In addition, during late adolescence, new behavioural patterns and life skills are formed and this is also a period where they make many decisions on their own for the first time.^35^ This, however, differed from findings of the studies in Tanzania and Bangladesh where the combination of risk factors for NCDs significantly declined with increasing adolescent age.^29,39^ This highlights the fact that no age group is spared from risk factors for NCDs.

More males had a higher proportion of combined behavioural risk factors compared to females in this present study, though the association was not statistically significant. This was similar to the findings from the study among school-going adolescents in Nepal.^40^ This differed from the findings from the study among adolescents in Vietnam and Brazil where more clustering of risk behaviours occurred in females than males.^5,14^ Variation in findings highlight that no age group or gender is spared from this public health threat.

These findings depict a grave public health concern for the prevention and control of NCDs. The combination of NCD risk factors in adolescents is very disconcerting as it is evident that the interaction of behavioural and physical risk factors for NCDs is more harmful to health than if the individual risk factors occurred independently.^29^ The public health implication of this implies that the burden of NCDs is likely to increase in the future if targeted actions are not put in place. The WHO has recommended a multi-sectoral approach for combating NCDs.^41^

## Conclusion

This study identified that there is evidence of co-occurrence and clustering of risk factors for NCDs among adolescents. This shows that there is an urgent need to tackle NCD prevention and control among this critical population using a multi-sectoral approach.
